# Erratum: 53-attosecond X-ray pulses reach the carbon K-edge

**DOI:** 10.1038/s41467-017-01281-1

**Published:** 2017-10-02

**Authors:** Jie Li, Xiaoming Ren, Yanchun Yin, Kun Zhao, Andrew Chew, Yan Cheng, Eric Cunningham, Yang Wang, Shuyuan Hu, Yi Wu, Michael Chini, Zenghu Chang

**Affiliations:** 1 Institute for the Frontier of Attosecond Science and Technology, CREOL, University of Central Florida, Orlando, FL 32816 USA; 2 Beijing National Laboratory for Condensed Matter Physics, Institute of Physics, Chinese Academy of Sciences, Beijing, 100190 China; 3 Department of Physics, University of Central Florida, Orlando, FL 32816 USA

This Article contains an error in the final two sentences of the section entitled “53-attosecond pulses retrieved by PROOF method”. These sentences should read: ‘Knowing the relative intensity change between the ungated and gated HHG, as well as the tin filter transmission, the photon flux for the 53 as pulse is estimated to be ~ 5 × 10^5^ photons per laser shot. The photon flux above carbon K-edge (284 eV) is >1 × 10^4^ photons per laser shot’.

