# Supplementary material for: 53-attosecond X-ray pulses reach the carbon K-edge
Source: Nat Commun. 2017 Aug 4;8:186. doi: 10.1038/s41467-017-00321-0 (PMC5543167; doi:10.1038/s41467-017-00321-0)
Supplement: Supplementary file 1 — Supplementary Information [file 41467_2017_321_MOESM1_ESM.pdf]

Description of Supplementary Files

File name: Supplementary Information
Description: Supplementary figures, supplementary notes and supplementary references.

File name: Peer review file

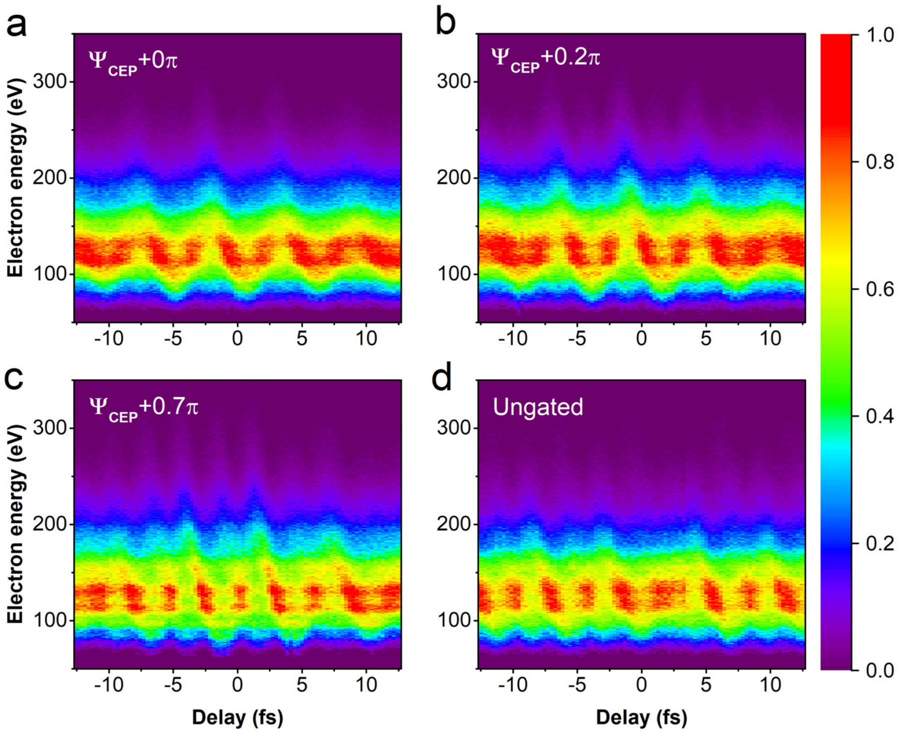

Supplementary Figure 1: Carrier-envelope phase influence on polarization gating. Experimental streaked photoelectron spectrograms using polarization gating (gate width δt_{G1}=3.6 fs) under different carrier-envelope phase (CEP) values (**a** to **c**), with the ungated case (**d**) shown for comparison. The fundamental IR field was filtered out using 100 nm tin filter for all cases.

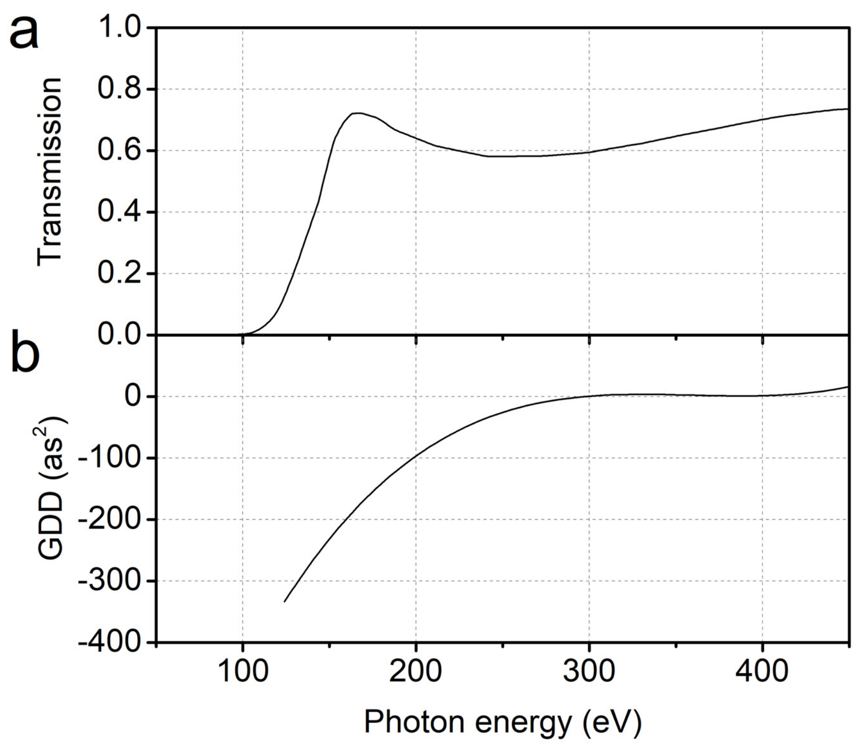

Supplementary Figure 2: Transmission and group delay dispersion of tin filter. Calculated transmission (a) and group delay dispersion (GDD) (b) of 100 nm tin filter.

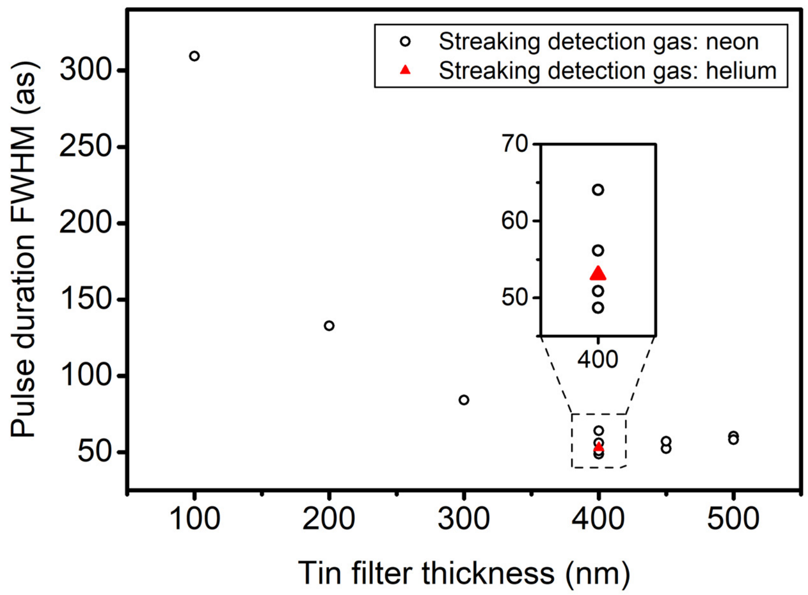

Supplementary Figure 3: Atto-chirp compensation by tin filter. PROOF-retrieved attosecond pulse duration with dipole correction at different filter thicknesses.

Supplementary Note 1: Laser system

The experiments are performed with an optical parametric chirped pulse amplifier (OPCPA) system at a 1 kHz repetition rate [1]. The front-end pump laser for the OPCPA is a home-built Ti:Sapphire chirped pulse amplifier (CPA) system with synchronized output of 2.2 mJ, 30 fs pulses and 18 mJ, 5 ps pulses. The 2.2 mJ pulses are focused into a neon-filled hollow-core fiber for white light generation. The white light pulses (0.5–0.9 μm) are compressed to 7 fs using chirped mirror pairs and then focused into a BIBO crystal for intra-pulse difference-frequency mixing [2]. This yields a broadband IR (1.2–2.2 μm) seed pulse with passive CEP stability. These IR seed pulses are stretched to 4.4 ps using an acousto-optic programmable dispersive filter (AOPDF) [3] and amplified inside three BIBO-based OPCPA stages, which are pumped by the synchronized output of the 18 mJ, 5 ps pulses from the Ti:Sapphire CPA system. The amplified IR pulses are compressed to 12 fs using the material dispersion of bulk fused silica. We use the AOPDF to fine-tune the dispersion and CEP of the IR pulses. The available IR pulse energy delivered to the experiments was more than 1.5 mJ.

Supplementary Note 2: Polarization gating for generating isolated attosecond pulses

The polarization gating setup in this experiment consists of two birefringent plates [4]. The optical axis of the first quartz plate is set to 45° with respect to the polarization of the incoming driving pulse. A delay T_d is introduced between the o- and e-pulses due to the refractive index difference between the o- and e-axes of quartz. The value of T_d can be controlled by changing the thickness of the quartz plate. The second zero-order quarter wave plate (optical axis set at 0°) converts the o- and e-pulses into counter-rotating circularly-polarized pulses. The electric field becomes linearly-polarized in the middle of the overlapped region and elliptically-polarized elsewhere. Effective HHG is only allowed in the linear portion of the pulse with a polarization gate width given by [5]:

$$\delta t_G = \varepsilon_{th} \tau_p^2 (\ln(2) T_d)^{-1}$$

where ε_{th}=0.1 is the threshold ellipticity for HHG using our driving field [6] and τ_p=12 fs is the pulse duration of our driving field.

We tested the polarization gate choosing $T_{d1}=T_0$, where T_0=5.7 fs is the laser optical period. This leads to a gate width δt_{G1}=3.6 fs>$T_0/2$. Since the HHG process repeats every half laser cycle for linearly-polarized driving field, a gate width $\delta t_{G1}>T_0/2$ could lead to more than one attosecond burst. We recorded streaked photoelectron spectrogram using polarization gating of δt_{G1}=3.6 fs at different CEP values and compared with ungated condition (Supplementary Fig. 1).

The streaked photoelectron spectrogram in Supplementary Fig. 1a shows a clear sinusoidal energy shift with a period of T_0. Upon changing the relative CEP value by only 0.2 π, half-cycle streaking becomes obvious in the upward and downward side of the energy shift (Supplementary Fig. 1b). Such a half-cycle energy shift is a signature of double attosecond pulses generated inside the polarization gate [7]. An even stronger half-cycle energy shift is observed when further changing the relative CEP value (Supplementary Fig. 1c). Such half-cycle energy shift would lead to a significant Fourier component $I_{2\omega}(v,\tau)$ to the photoelectron spectrogram. In order to suppress the double pulses, we applied polarization gating using a shorter gate width δt_{G2}=1.8 fs<T_0/2 with optimized CEP values. The resulted 2ω component $I_{2\omega}(v,\tau)$ is more than 10 times smaller than the ω component $I_\omega(v,\tau)$, which confirms the presence of an isolated attosecond pulse for all streaking experiments reported in this paper.

Supplementary Note 3: Atto-chirp compensation and error bar for streaking measurement

It is known that the attosecond pulses generated by HHG are not transform-limited in their temporal duration [8]. At the microscopic level, two quantum trajectories of the laser-driven electron contribute to the high-harmonic pulse emission. Typically only the short trajectory is phase-matched in experiments. The chirp is positive for emissions from the short trajectory, which can potentially be compensated by the negative dispersion of a tin filter in the region of 100 to 300 eV [9]. However, the group delay dispersion (GDD) of tin approaches zero at 300 eV (Fig. S2), meaning that atto-chirp compensation is only effective for the lower energy part (< 200 eV) of the spectrum.

Supplementary Fig. 3 shows the retrieved attosecond pulse durations versus the tin filter thickness. Neon gas was first used as the detection gas for its relatively-large absorption cross-section compared to helium. We combined several tin filters to find the optimum thickness for atto-chirp compensation. A clear trend of decreasing pulse durations is found as the filter thickness increases from 100 to 400 nm. Further increasing filter thickness to 500 nm led to an overcompensated atto-chirp on the low energy side, resulting in a slightly increased pulse duration. Multiple measurements are repeated at 400 nm, 450 nm and 500 nm thickness to confirm the experimental repeatability. At a thickness of 400 nm, a total of 5 measurements are performed and presented. A standard deviation of 6 as (calculated from the 5 results at 400 nm) is used for the error bar in Fig. 3 in the main text, which represents the repeatability of our measurement.

Supplementary Note 4: Retrieval procedure in PROOF

In PROOF, the spectrum and phase of the attosecond photoelectron burst are retrieved using an iterative algorithm, the goal of which is to minimize the error between the component of the experimental spectrogram oscillating with the laser frequency ($I_\omega(v,\tau)$ in Eq. (1) of the main text), and a guessed trace, given by Eq. (11) in Ref. 11. $I_\omega(v,\tau)$ can be written as the product of two components: a modulation amplitude $\gamma(v)$ and the laser-frequency oscillation component

$sin[\omega\tau+\alpha(v)]$, where $\alpha(v)$ describes the phase of the oscillation. Both $\gamma(v)$ and $\alpha(v)$ epend on (and can be calculated from) the spectral amplitude and phase of the attosecond burst.

In the algorithm, the error between the experimental and guessed $\gamma(v)$ and $\alpha(v)$ are separately minimized, in an iterative fashion. The algorithm proceeds as follows:

Initialization: Let the spectral amplitude of the attosecond photoelectron burst be given by $U(v)=(I_o(v))^{1/2}$, where $I_o(v)$ is the DC Fourier component of the experimental spectrogram. This is a very accurate guess in the case where the streaking intensity is low; however, it must be refined by further iteration (spectrum optimization) under practical experimental conditions. To avoid introducing noise into the algorithm, the spectrum is smoothed using a cubic spline fitting function.

Phase optimization: Guess the spectral phase of the attosecond burst $\phi(v)$ which minimizes the error function between the experimentally-obtained phase angle $\alpha(v)$ and that calculated from the guessed $U(v)$ and $\phi(v)$. For this minimization, the value of the phase at each electron energy is allowed to vary between 0 and 2π, and the spectral phase function $\phi(v)$ is obtained by unwrapping the phase and applying a cubic spline fitting before evaluating the error function, in order to avoid introducing noise into the algorithm.

Spectrum optimization: Guess the spectral amplitude of the attosecond burst $U(v)$ which minimizes the error function between the experimentally-obtained modulation amplitude $\gamma(v)$ and that calculated using the guessed $U(v)$ and $\gamma(v)$. For this minimization, the value of the amplitude is allowed to vary freely, and the spectral amplitude function $U(v)$ is obtained by applying a cubic spline fitting before evaluating the error function, in order to avoid introducing noise into the algorithm.

Repeat phase and spectrum optimization iteratively. After each iteration, evaluate the error function between the experimentally obtained laser-frequency filtered spectrogram $I_\omega(\nu,\tau)$ and that calculated using the guessed $U(v)$ and $\phi(v)$. Once the error has decreased to a suitable value, stop the loop and export the guessed $U(v)$ and $\phi(v)$. Apply the dipole correction to the guessed $U(v)$ and $\phi(v)$ to obtain the attosecond photon pulse spectrum and phase.

The metrics used to evaluate the accuracy of the retrieval are therefore the agreement between the filtered spectrogram $I_\omega(v,\tau)$ and that calculated from the guessed $U(v)$ and $\phi(v)$, as well as the agreement between the measured and guessed photoelectron spectrum. The agreement in Fig. 3(b) is excellent, with the only discrepancy occurring in the photoelectron spectral region around 225 eV, where the experimental modulation amplitude drops to nearly zero, and the phase angle cannot be accurately extracted.

Supplementary References

1. Y. Yin, J. Li, X. Ren, K. Zhao, Y. Wu, E. Cunningham, Z. Chang, High-efficiency optical parametric chirped-pulse amplifier in BiB_3O_6 for generation of 3 mJ, two-cycle, carrier-envelope-phase-stable pulses at 1.7 μm. *Opt. Lett.* **41**, 1142-1145 (2016).

2. T. Fuji, A. Apolonski, F. Krausz, Self-stabilization of carrier-envelope offset phase by use of difference-frequency generation. *Opt. Lett*. **29**, 632 (2004).

3. P. Tournois, Acousto-optic programmable dispersive filter for adaptive compensation of group delay time dispersion in laser systems. *Optics Commun*. **140**, 245 (1997).

4. I. J. Sola, E. Mével, L. Elouga, E. Constant, V. Strelkov, L. Poletto, P. Villoresi, E. Benedetti, J.-P. Caumes, S. Stagira, C. Vozzi, G. Sansone, M. Nisoli, Controlling attosecond electron dynamics by phase-stabilized polarization gating. *Nat. Phys*. **2**, 319-322 (2006).

5. V. Strelkov, A Zaïr, O. Tcherbakoff, R. López-Martens, E. Cormier, E. Mével, E. Constant. Single attosecond pulse production with an ellipticity-modulated driving IR pulse. *J. Phys. B* **38**, L161 (2005).

6. J. Li, X. Ren, Y. Yin, Y. Cheng, E. Cunningham, Y. Wu, Z. Chang, Polarization gating of high harmonic generation in the water window. *Appl. Phys. Lett.* **108**, 231102 (2016).

7. J. Gagnon, V. S. Yakovlev, The robustness of attosecond streaking measurements. *Opt. Express* **17**, 17678 (2009).

8. Y. Mairesse, A. De Bohan, L. Frasinski, H. Merdji, L. Dinu, P. Monchicourt, P. Breger, M. Kovačev, R. Taïeb, B. Carré, Attosecond synchronization of high-harmonic soft x-rays. *Science* **302**, 1540-1543 (2003).

9. D. H. Ko, K. T. Kim, C. H. Nam, Attosecond-chirp compensation with material dispersion to produce near transform-limited attosecond pulses. *J. Phys. B* **45**, 074015 (2012).
